# Mutations in the nuclear localization sequence of the *Aristaless *related homeobox; sequestration of mutant ARX with IPO13 disrupts normal subcellular distribution of the transcription factor and retards cell division

**DOI:** 10.1186/1755-8417-3-1

**Published:** 2010-01-05

**Authors:** Cheryl Shoubridge, May Huey Tan, Tod Fullston, Desiree Cloosterman, David Coman, George McGillivray, Grazia M Mancini, Tjitske Kleefstra, Jozef Gécz

**Affiliations:** 1 Department of Genetics and Molecular Pathology, SA Pathology at the Women's and Children's Hospital, North Adelaide, South Australia 5006, Australia; 2 Department of Paediatrics, University of Adelaide, Adelaide, South Australia 5001, Australia; 3 Department of Metabolic Medicine, The Royal Children's Hospital, Brisbane, Queensland 4029, Australia; 4 Genetics Health Services Victoria, Murdoch Children's Research Institute, Melbourne, Victoria 3052, Australia; 5 Department of Clinical Genetics, Erasmus University Medical Centre, Rotterdam 3015GE, The Netherlands; 6 Department of Human Genetics, Radboud University Nijmegen Medical Centre, Nijmegen, The Netherlands

## Abstract

**Background:**

*Aristaless *related homeobox *(ARX) *is a paired-type homeobox gene. ARX function is frequently affected by naturally occurring mutations. Nonsense mutations, polyalanine tract expansions and missense mutations in *ARX *cause a range of intellectual disability and epilepsy phenotypes with or without additional features including hand dystonia, lissencephaly, autism or dysarthria. Severe malformation phenotypes, such as X-linked lissencephaly with ambiguous genitalia (XLAG), are frequently observed in individuals with protein truncating or missense mutations clustered in the highly conserved paired-type homeodomain.

**Results:**

We have identified two novel point mutations in the R379 residue of the ARX homeodomain; c.1135C>A, p.R379S in a patient with infantile spasms and intellectual disability and c.1136G>T, p.R379L in a patient with XLAG. We investigated these and other missense mutations (R332P, R332H, R332C, T333N: associated with XLAG and Proud syndrome) predicted to affect the nuclear localisation sequences (NLS) flanking either end of the ARX homeodomain. The NLS regions are required for correct nuclear import facilitated by Importin 13 (IPO13). We demonstrate that missense mutations in either the N- or C-terminal NLS regions of the homeodomain cause significant disruption to nuclear localisation of the ARX protein *in vitro*. Surprisingly, none of these mutations abolished the binding of ARX to IPO13. This was confirmed by co-immunoprecipitation and immmuno fluorescence studies. Instead, tagged and endogenous IPO13 remained bound to the mutant ARX proteins, even in the RanGTP rich nuclear environment. We also identify the microtubule protein TUBA1A as a novel interacting protein for ARX and show cells expressing mutant ARX protein accumulate in mitosis, indicating normal cell division may be disrupted.

**Conclusions:**

We show that the most likely, common pathogenic mechanism of the missense mutations in NLS regions of the ARX homeodomain is inadequate accumulation and distribution of the ARX transcription factor within the nucleus due to sequestration of ARX with IPO13.

## Background

The genes on the X-chromosome contribute significantly to genetic aetiology of intellectual disability [1]. The *Aristaless *related homeobox gene *(ARX) *[GenBank: NM_139058.2] is one of the more frequent contributors [1]. ARX belongs to a subset of *Aristaless*-related Paired-class (Prd-class) homeodomain proteins [2] and contains multiple domains, including the *aristaless *domain, homeodomain, the octapeptide and 4 polyalanine tracts [3]. *ARX *mutations cause intellectual disability with or without additional features including epilepsy, infantile spasms, dystonia, lissencephaly, autism and dysarthia [3-5]. To date, over 90 families and individual cases with 40 different types of mutations have been reported. These include missense mutations, protein truncations but most frequently, polyalanine tract expansions [6-19].

When *Arx *was first ablated in mouse [5], the phenotype recapitulated many clinical aspects of X-linked lissencephaly with ambiguous genitalia [XLAG; MIM 300215] [20,21]. Subsequently, this led to the identification of *ARX *mutations in patients with XLAG [5]. Lissencephaly is one of a heterogenous group of disorders arising from aberrant neuronal migration. The characteristic 'smooth brain', due to a paucity of normal gyri and sulci, is due to either the arrest of neuronal migration (classical) or an over-migration of neurons (cobblestone). Mutations in the X-linked gene *DCX *(MIM 300121) [22] and four autosomal genes: *LIS1 *(MIM 601545) [23], *RELN *(MIM 600514) [24], *TUBA1A *(MIM 602529) [25,26] and *VLDLR *(MIM 192977) [27] have been associated with distinct lissencephaly syndromes. However, mutations in *ARX *are the only identified genetic cause underlying the distinct syndrome of XLAG. This syndrome differs from the other forms of lissencephaly, displaying a thickened cortex with posterior to anterior gradient of gyral malformation, agenesis of the corpus callosum and ambiguous genitalia [28].

There are currently 31 families reported with XLAG phenotypes, with and without additional features, due to 27 different mutations in *ARX*. The majority of these families are predicted to arise from protein truncation and the loss-of-function of the mature ARX protein [5,7,9,12,28-31]. In the remaining families with XLAG, single nucleotide substitutions clustered in the homeodomain or, in one case, just prior to the *aristaless *domain, are predicted to give rise to amino acid substitutions in the mature protein. Several of these single nucleotide substitutions occur in residues of nuclear localization sequences (NLS) that flank both ends of the ARX homeodomain. In the N-terminal NLS (NLS2) there are four naturally occurring point mutations - R332P, R332H and R332C which cause XLAG and a residue adjacent to this arginine, T333N, which causes Proud syndrome (agenesis of the corpus callosum with ambiguous genitalia) [ACC/AG; MIM 30004]. We have recently identified novel point mutations in a single residue of the NLS3 region flanking the C-terminal portion of the homeodomain, R379. In one case, a substitution of R379 with lysine (L) has been identified in a patient presenting a phenotype of XLAG (D Coman, unpublished data). In contrast, a second change in the same residue resulting in the substitution of R379 with serine (S) was identified in a proband and his female relatives [32]. Interestingly, the phenotype includes infantile spasms and severe mental retardation without obvious brain malformation.

The basic residues of ARX NLS2 and NLS3 are important for the correct localization of ARX in the nucleus through interaction with Importin 13 (IPO13) [33]. IPO13 is a member of the importin-β superfamily involved in nuclear import and export of a variety of proteins [34,35]. In addition to IPO13, a recent study suggests that multiple importins are utlized to import murine Arx into the nucleus, including importin β1 [36]. Regardless of the importin utilized, the driving force for nuclear protein import is provided by RanGTP and its interaction with the importin-cargo complex [37]. We show that missense mutations of NLS2 and NLS3 cause significant disruption to the nuclear localization of ARX *in vitro*. However, these mutations do not abolish the binding of ARX to IPO13. Interestingly, the binding of mutant ARX protein to endogenous IPO13 is indistinguishable from the binding to N-terminally truncated IPO13 lacking the RanGTPase binding domain. Hence, the ability of the mutant ARX-IPO13 complex to uncouple in the RanGTP rich nuclear environment appears to be compromised. As part of our investigation into the pathogenic mechanism underlying these mutations we identify TUBA1A, a component of the cytoskeleton important in mitosis, as a novel protein partner for ARX and examine the impact of sequestration of mutant ARX cargo with IPO13 on mitosis and cell division.

## Materials and methods

### Subjects and families

The relevant research ethics committees and institutional review boards of collaborating institutions approved this study. Clinical information and DNA samples were collected with informed consent.

### Cloning of mutant full-length *ARX *constructs

The cloning of the full-length human *ARX *cDNA construct in pCMV-Myc vectors (ARX-Wt; 1-562 aa) and the V5-IPO13 (aa 217-963; minus the RanGTPase binding site) have been described previously [33]. Single nucleotide substitutions were introduced into the pCMV-Myc-ARX-Wt full-length construct via site directed mutagenesis (Stratagene) following the manufacturer's instructions. The mutations included c.995G>C leading to p.R332P (R332P) in the NLS2 region, c.998C>A leading to p.T333N (T333N) close to NLS2, c.1136G>T leading to p.R3479L (R379L) and c.1135C>A leading to p.R379S (R379S) in the NLS3 region. A double mutant construct was engineered with a mutation in each the NLS2 (c.995G>C) and NLS3 (c.1136G>T) regions in the same cDNA construct (R332P-R379L). In addition, a construct containing a c.1058C>T change leading to p.P353L (P353L) was engineered to provide an ARX mutation located within the homeodomain but outside of the NLS regions. All clone preparations were verified by sequencing of the entire coding region.

### Cell culture and transient transfection

HEK293T cells were maintained in Dulbecco's modified Eagle's medium supplemented with 10% (v/v) fetal calf serum. All cells were cultured in the presence of 100 U/ml sodium penicillin and 100 μg/ml of streptomycin sulphate in 5% CO_2 _at 37°C. Cells plated at 4 × 10^5 ^per well in a six- well plate the day before transfection in media lacking antibiotics. Routinely, cells were transfected with a total of 1 μg of plasmid DNA using Lipofectamine 2000 (Invitrogen, CA, USA) following the manufacturer's instructions.

### Antibodies

The following antibodies were used for immunofluorescence and/or western immunoblot analysis: mouse anti-ARX antibody (2 μg/ml final); goat anti-IPO13 (1:500) (Imgenex, CA, USA); rabbit-anti-alpha-tubulin (1:1500) (Rockland, PA, USA); rabbit anti-V5 antibody (1:5000) (Bethyl Laboratories, Tx, USA). The secondary antibodies were either fluorescent labelled; goat anti-mouse-IgG conjugated to CY3 (1:1000) (Jackson Laboratories, Maine, USA); goat anti-mouse-IgG conjugated to FITC (1:1000) (Dako, Glostrup, Sweden); goat anti-rabbit-IgG Alexa Fluor 488 (1:800) (Invitrogen); donkey anti-goat-IgG Alexa Fluor 555 (1:1000) (Invitrogen) or horseradish-peroxidase (HRP) conjugated; mouse anti-Myc HRP conjugated antibody (1:5000) (Invitrogen); mouse anti-V5 HRP conjugated antibody (1:5000) (Invitrogen); goat anti-mouse-HRP antibody (1:1000) (Dako); goat anti-rabbit HRP antibody (1:1000) (Dako); rabbit anti-goat HRP (1:2000).

### Immunofluorescence and microscopy

Transfected cells were harvested at 24 and 48 h post-transfection by fixation in 3.7% formaldehyde-phosphate buffered saline (PBS) (v/v) and permeabilized in 0.2% (v/v) triton PBS for 5 min. Blocking of non-specific binding of the secondary antibody was achieved routinely by addition of 5% skim-milk powder (w/v) in Tris buffered saline and 0.5% Tween (v/v) (TBS-T) before incubation of the primary and secondary antibodies diluted in 1% milk (w/v) in TBS-T. Removal of excess block and antibody was achieved with multiple washes of TBS-T. Nuclei were counterstained with DAPI (Molecular probes, Invitrogen).

For subcellular localization studies: Between 1000-4000 transfected cells were counted for each construct from at least three different transfection reactions using standard fluorescence microscopy. The percent of transfected cells with abnormal localization or aggregates was determined as the number of cells containing abnormal localization or aggregates divided by the total number of ARX positive cells. Sub-cellular localization images were captured by Leica TCS SP5 spectral confocal microscope using a 100× Plan apochromat objective. Z-stacks were taken at 0.25 μm intervals and maximal projections were made for all cell images.

For mitosis studies: Between 1200 and 4000 cells were analysed at 24, 48 and 72 h post-transfection for: (i) percentage of cells transfected; (ii) percentage of mitotic cells in both transfected and un-transfected cells; and (iii) and the phase of mitosis for each cell undergoing division.

### Yeast-2 hybrid screening

Plasmids encoding the *ARX *homeodomain (ARX-HD) were fused to the GAL4 DNA binding domain and the library protein were fused to GAL4-activation domain. The MaV203 yeast strain was co-transformed with the *ARX-HD *construct and human fetal brain cDNA library (ProQuest, Invitrogen). Colonies were selected on media lacking leucine, tryptophan and histidine and positive clones were analysed for expression of three reporter genes (*HIS3*, *URA3 *and *LacZ*) by measuring β-galactosidase activity. Library inserts of positive clones that activated more than one reporter gene were amplified by polymerase chain reaction and sequenced to determine the identity of the library clone.

### Co-immunoprecipitation

Cells transfected with *Myc-ARX*, both with and without *V5-IPO13 *were harvested at 24 hrs post-transfection and cell lysates prepared using lysis buffer (120 mM NaCl, 50 mM Tris-HCl (pH 8.0), 0.5% NP-40 (v/v), 1× protease inhibitor cocktail (Sigma, AZ, USA), 1 mM Na_3_VO_4_, 1 mM NaF, 1 mM PMSF). Lysates were clarified by centrifugation (15 min, 13,000 *g *at 4°C). Aliquots of extracts were immunoprecipitated (IP) overnight at 4°C. Protein-A sepharose was pre-treated with un-transfected HEK293T cell lysate to ameliorate non-specific binding of cell proteins. The IP reactions were incubated with the pre-treated protein-A sepharose for 1 h at 4°C before removal of non-specifically bound proteins with four changes of high stringency wash buffer (500 mM NaCl, 20 mM Tris-HCl (pH 8.0), 1 mM EDTA, 0.5% NP-40 (v/v)) to ensure adequate removal of non-specific binding of alanine tract containing ARX protein. Proteins bound to the protein-A sepharose were eluted in SDS loading buffer (62.5 mM Tris-HCl (pH 6.8), 2% SDS (v/v), 10% glycerol (v/v), 5% β-Mercaptoethanol (v/v), 0.001% bromophenol blue (w/v), heated to 65°C before addition and incubated for 3 min). IP proteins were subjected to SDS-PAGE and transferred to nitrocellulose membrane. Lysates from HEK293T cells producing either Myc-ARX alone or V5-IPO13 alone were used as controls.

In co-transfected cells; Myc-ARX protein was IP with 0.5 μg of anti-Myc antibody (Santa Cruz Biotech, CA, USA) or the reciprocal co-immunoprecipitation (Co-IP) with 0.5 μg of rabbit anti-V5 antibody immunoprecipitating V5-IPO13 protein. IP proteins were analysed for the presence of Myc-ARX and V5-IPO13 by western immunblotting. In cells transfected with ARX alone, endogenous IPO13 was IP with 1 μg of goat anti-IPO13 and Co-IP of Myc-ARX protein was identified using mouse anti-Myc HRP conjugated antibody.

To verify the interaction between Myc-ARX and TUBA1A, HEK293T cells transfected with Myc-ARX constructs were lysed and immunoprecipitated with mouse anti-Myc antibody. IP proteins were analysed for the presence of Myc-ARX and endogenous alpha-tubulin by western immunoblotting.

### Statistical analysis

All data are reported as mean ± standard error of mean, determined from a minimum of three separate transfection reactions, with the number of cells counted indicated in each figure legend. Differences in the percentage of transfected cells with abnormal subcellular localization were analysed by Kruskal-Wallis Test and when significance was reached a one-tailed pair-wise comparison was achieved using Wilcoxon-Mann-Whitney U test. In the case of the mitosis data, differences in the proportions of cells in various phases of mitosis at increasing times post-transfection were compared by two-way ANOVA, with mutations and time as factors. *P *< 0.05 was considered significant.

## Results

### Novel mutations in ARX homeodomain

**Figure 1:**
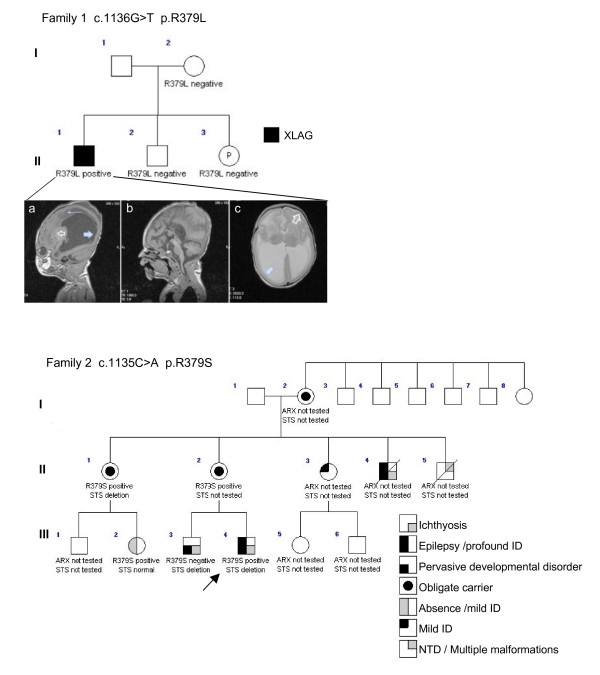
**Pedigree of families with novel mutations in the ARX homeodomain**. Open symbols represent normal individuals, filled symbols represent affected individuals as per the key. Proband indicated with arrow. Individual generations are numbered with Roman numerals. MRI findings in proband of family 1 (a) sagittal T1 MR sequence demonstrates lissencephaly P > A and marked lateral ventriculomegaly. Note subependymal cysts (large arrow) abnormal periventricular white matter signal (small arrow) and abnormal basal ganglia signal clear arrow, (b) sagittal T1 MR sequence of the midline demonstrates agenesis of the corpus callosum and (c) axial T2 MR sequence demonstrates colpocephaly and lissencephaly with transition from posterior agyria (solid arrow) to anterior pachygyria (open arrow).

We have identified two novel point mutations in the same residue of the NLS3 region of ARX homeodomain in two unrelated families: (i) An apparently *de novo *and novel single base substitution c.1136G>T in exon 4 of *ARX*, resulting in the substitution of an arginine to a leucine amino acid substitution at position 379 (R379L); and (ii) A novel single base substitution c.1135C>A in exon 4 of *ARX *was identified in the proband and three female relatives [32], resulting in the substitution of an arginine to a serine amino acid substitution at position 379 (R379S) (Figure 1). In the first case a clinical diagnosis of XLAG phenotype included mildly dysmorphic features, ambiguous genitalia with a micropenis, fused scrotum, cryptorchidism and a severe refractory seizure disorder. Magnetic resonance imaging (MRI) identified lissencephaly with agyria posteriorly transitioning in the mid-parietal region to pachygyria anteriorly, agenesis of the corpus callosum and a severe encephalopathy (Figure 1a-c). Other clinical findings include hypothalamic dysfunction, hypophosphatasia and severe chronic diarrhoea with evidence of pancreatic insufficiency and small bowel malabsorption. No developmental milestones were attained and the disease was ultimately lethal. The c.1136G>T mutation was not found in the mother of the patient and her MRI brain scan showed a normally formed corpus callosum (data not shown). In the second case, the associated phenotype of carrier females of the c.1135C>A mutation from the family were reported [32]. We report here the clinical findings of the male proband, who had a history of infantile spasms and severe intellectual disability (Figure 1 Family 2, patient III-4). He was born after a pregnancy of 39 weeks by spontaneous delivery with a birth weight of 2600 g. In the first months eye contact was not adequate. At the age of 6 months he experienced seizures with tonic extension of the arms and turning of the eye globes, which progressively increased in frequency and were followed by apnoea. The electroencephalograph showed a disorganized background pattern compatible with hypsarrhythmia. On examination he was apathetic, made no eye contact and showed insufficient spontaneous movements. His head growth progressively slowed and at 6 years of age his occipitofrontal circumference was below -2SD. He had no significant psychomotor development and at 5 years of age had spastic tetraparesis with truncal hypotonia. At 8 years of age he is wheelchair bound, makes no eye contact, reacts to touch and can chew and swallow food. He has recurrent airway infections. His seizures are refractory to medical treatment, notwithstanding the use of several antiepileptic drug medications. Interestingly, this patient showed no XLAG or brain malformation when examined by MRI (data not shown). The patient had unilateral cryptorchidism. The presence of dry scaly skin prompted testing for steroid sulphatase deficiency and the diagnosis was confirmed by genomic DNA analysis that showed an STS gene deletion and leukocytes enzyme assay that showed reduced activity (data not shown). The borders of the STS deletion were fine mapped in another family member with the same deletion by 250 K SNP arrays and were comprised between SNP_A-2122915 and SNP_A-1933813 on chromosome Xp22.

### Missense mutations in NLS2 and NLS3 disrupt nuclear localization of ARX

**Figure 2:**
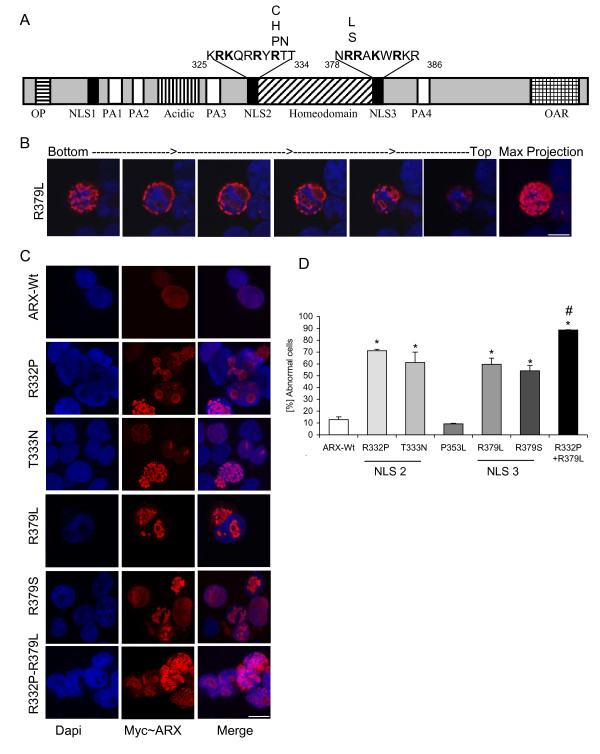
**Missense mutations in nuclear localization sequence (NLS) regions of the *Aristaless related homeobox *(ARX) homeodomain disrupt the normal nuclear subcellular localization in HEK293T cells**. (A) Schematic of the human ARX protein. Known functional domains are highlighted in the open reading frame: Octapeptide (OP) as horizontally hatched rectangle, NLS as three black rectangles, polyalanine tracts (PA) as four white rectangles, acidic domain as vertically hatched rectangle, homeodomain crosshatched and *Aristaless *domain (OAR) hatched. The sequences flanking the homeodomain are shown above the black rectangle, the locations indicated at either end, with the basic residues that are part of the NLS-like motifs in bold. (B) Individual focal planes of cell transfected with Myc-R379L (NLS3) mutation construct show aggregates of mutant ARX protein form both inside the nucleus and outside in the cytoplasm surrounding the nucleus. Panel on far left is at the top of the cells (focal plane 2) with subsequent images an additional four focal planes further through the cell, maximal projection of all images is shown on the far right. The aggregates often displace the nuclear material, shown by distortion and absence of the blue DAPI signal in location of the aggregates. Scale bar = 10 μM. (C) Representative pictomicrographs of the localization of the Wt and mutant ARX protein 24 hrs post transfection. My~ ARX detected by anti-ARX Ab and Cy3 conjugated secondary antibody (left panel) merged with DAPI stained nuclear material (right panel). Scale bar = 10 μM. (D) The percentage of transfected cells displaying abnormal localization as inclusions or aggregates in the nucleus, with or without aggregates in the cytoplasm, was determined from between 1000 and 4000 transfected cells per construct, from at least two separate transfection reactions, 24 h post-transfection. Full-length Myc-tagged constructs transfected are listed along the bottom of the graph; ARX Wt (open bar), R332P (light grey bar), T333N (grey bar), P353L mutant (cross hatched bar), R379L and R379S (both dark grey bars) and double mutant R332P-R379L (black bar). All groups comparison of the percentage of transfected cells with abnormal subcellular localization of expressed ARX protein was achieved by parametric Kruskal-Wallis test. * *P *< 0.05 versus ARX-Wt, ^# ^versus ARX-NLS mutants with single aa substitutions.

Transient transfection of full length Myc-tagged ARX-Wt and mutants were conducted to examine changes in subcellular localisation of the mutant proteins compared to the ARX-Wt protein. We tested R332P, T333N, R379L and R379S mutations (Figure 2A). We used confocal microscopy to analyse subcellular localisation, highlighted for the R379L mutation (Figure 2B). Aggregates are clearly distinguished in the peri-nuclear region of the cytoplasm, with a large aggregate mass in the top right quadrant. In addition to the cytoplasmic localisation, a series of aggregates in the nuclear space can be identified. The percentage of transfected cells with aberrant sub-cellular localisation of ARX protein was assessed in at least 1000 transfected cells from a minimum of three separate transfection experiments. For counting purposes, transfected cells were categorised as (1) normal localization; diffuse staining restricted to the nucleus (Figure 2C, top panel) or (2) abnormal localization, aggregates of mutant protein in either the nucleus or in both the nucleus and cytoplasm (Figure 2C). Expression of the Myc-tagged ARX-Wt protein was detected in a non-homogenous pattern across the nucleus with little or no expression detectable in the cytoplasm. We observed this normal pattern of staining in over 87% of cells transfected with ARX-Wt at 24 hrs (Figure 2D) and 91% of cells at 48 h post-transfection (data not shown). Transient over-expression leads to aberrant localization of the ARX-Wt protein in approximately 13% of transfected cells (Figure 2D), generally in the form of a single bright spot in the nucleus in addition to the normal diffuse staining (data not shown). In contrast, ARX NLS mutant proteins were found within the nucleus as either multiple, small aggregates, or longer continuous 'ribbon like structures' or larger aggregates in which the DAPI stained nuclear material had been excluded. The mutant protein was also found in aggregates in the cytoplasm, often forming large bodies to one side of the nucleus distorting the DAPI stained nuclear material (Figure 2C). Routinely, cells with abnormal localization of the mutant protein were a mixture of cells with aggregates in the nucleus and no detectable expression of ARX in the cytoplasm, in addition to cells with aggregates of mutant ARX in both the nucleus and cytoplasm. No cells with ARX protein exclusively in the cytoplasm were noted. The percentages of transfected cells with abnormal ARX protein localization were significantly elevated for all NLS mutations tested compared to ARX-Wt transfected cells (Figure 2D). Mutations leading to severe clinical outcomes resulted in the highest levels of cells with abnormal protein localization. Over-expression of R332P for 24 h resulted in 72% of transfected cells in which the mutant protein was incorrectly localized; an approx 5.5-fold increase compared to the 13% of ARX-Wt transfected cells. The T333N mutation, adjacent to the NLS2 region, mis-localized in 61% of transfected cells. Similarly, the R379L mutation resulted in 60% of all transfected cells with abnormal protein localization (Figure 2D). Unlike the other three mutations, the R379S mutaiton leads to a phenotype without malformation of the brain. In cells expressing the R379S mutation the increase in cells with abnormal localisation of mutant protein was the lowest of all the mutations tested at 54% (Figure 2D). In contrast to the NLS mutations, when another mutation the middle of the ARX homeodomain (P353L; XMESID phenotype) was expressed in HEK293T cells there were no increased levels of cells with mis-localized protein compared to ARX-Wt (Figure 2D). When a single construct containing two mutations (R332P and R379L) was transfected into HEK293T cells we found 90% of all of transfected cells displayed abnormal localisation of the mutant protein (Figure 2D). This increase was above the effect of either mutation alone and indicates the NLS regions flanking the homeodomain are not redundant but likely act co-operatively.

### Missense mutations in NLS2 and NLS3 do not abolish ARX interaction with N-terminally truncated IPO13

**Figure 3:**
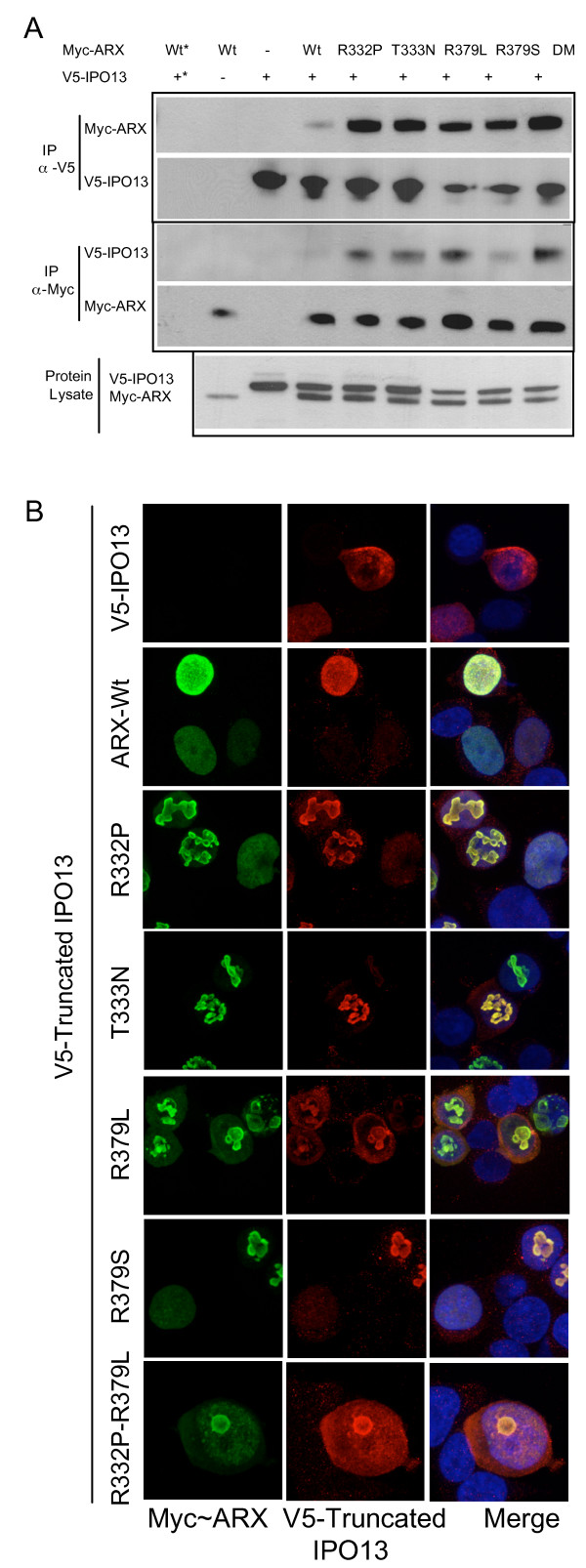
**NLS mutant ARX co-immunoprecipitates and co-localizes with N-terminally truncated IPO13 in mammalian cells**. (A) HEK293T cells transfected with Myc-ARX-Wt or NLS mutant constructs and V5-IPO13 were lysed and protein immunoprecipitated (IP) with antibodies against the V5 or Myc tags. Samples were loaded onto 4%-12% SDS-PAGE gels and analysed for the presence of co-IP proteins. Reciprocal Co-IPs was conducted on replicate samples; IP with rabbit anti-V5 antibody (top panel) and detection of Myc-ARX proteins bound to V5-IPO13 by immunoblotting with mouse anti-Myc HRP conjugated antibody. All samples transfected with V5-IPO13 showed a protein band of the correct size upon blotting with anti-V5 horseradish-peroxidase (HRP) conjugated antibody. IP with mouse anti-Myc antibody (middle panel) and detection of V5-IPO13 protein bound to Myc-ARX by immunblotting with mouse anti-V5 HRP conjugated antibody. All samples transfected with Myc-ARX showed a protein band of the correct size upon blotting with anti-Myc HRP conjugated antibody. Specific IP of each over-expressed protein was achieved with no band present in samples from cells transfected with both Myc-ARX and V5-IPO13 but no IP antibody added (*). Cells transfected with Myc-ARX alone or V5-IPO13 alone was used as negative controls. V5-IPO13 (~88.6 kDa) and Myc-ARX (~62.2 kDa) are both present in protein lysates (bottom panel). (B) Pictomicrographs showing V5-IPO13 co-localises with ARX-Wt and mutant ARX protein (indicated on left of panels) in both nuclear and cytoplasmic aggregates in cells co-transfected with Myc-ARX and V5-IPO13 constructs for 24 h. ARX detected by anti-ARX antibody and FITC conjugated secondary antibody (left panel), V5-IPO13 detected by anti-IPO13 antibody and Cy3 conjugated secondary antibody (middle panel) and merged image including blue DAPI stained nuclear material (right panel). Scale bar = 10 μM.

In order to test if ARX with mutations in NLS2 or NLS3 were capable of binding to IPO13 in the cell environment we co-transfected V5-tagged-IPO13 lacking the N-terminal RanGTPase binding site. This truncated V5-IPO13 protein can bind and transport the ARX cargo but is unable to disassociate the cargo upon reaching the RanGTP rich nuclear environment. We IP over-expressed truncated V5-IPO13 by anti-V5 antibody (Figure 3A, top box, second panel) and Myc-ARX by anti-Myc antibody (Figure 3A, middle box, second panel) from whole cell extracts, with no protein detected in parallel experiments in which no IP antibody was added (Figure 3A, lane 1). In cells expressing both Myc-ARX and V5-IPO13 we were able to detect co-IP of Myc-ARX protein when immunoprecipitating with anti-V5 antibody (Figure 3A, top box, first panel) and in the reciprocal immunoprecipitation with anti-Myc antibody we detected Co-IP of V5-IPO13 protein (Figure 3A, middle box, first panel). Regardless of the antibody used for pull down we observed a stronger Co-IP signal for all ARX mutant samples, including the ARX protein containing the double NLS mutation compared to the ARX-Wt protein. In agreement with the Co-IP data, there was a shift in the truncated V5-IPO13 signal from diffuse cytoplasmic localisation when transfected alone (Figure 3B top panel) to complete overlap with the nuclear expression of ARX-Wt protein (Figure 3B second panel) and nuclear and cytoplasmic expression of NLS2 and NLS3 mutant ARX signal in co-transfected cells (Figure 3). These results were consistent across all of the mutations tested, including the R332P-R379L mutant. These results clearly indicate the mutant ARX proteins were able to bind truncated V5-IPO13, even when there were single amino acid substitutions in both NLS regions.

### Endogenous IPO13 binds to ARX protein containing missense mutations in NLS2 and NLS3

**Figure 4:**
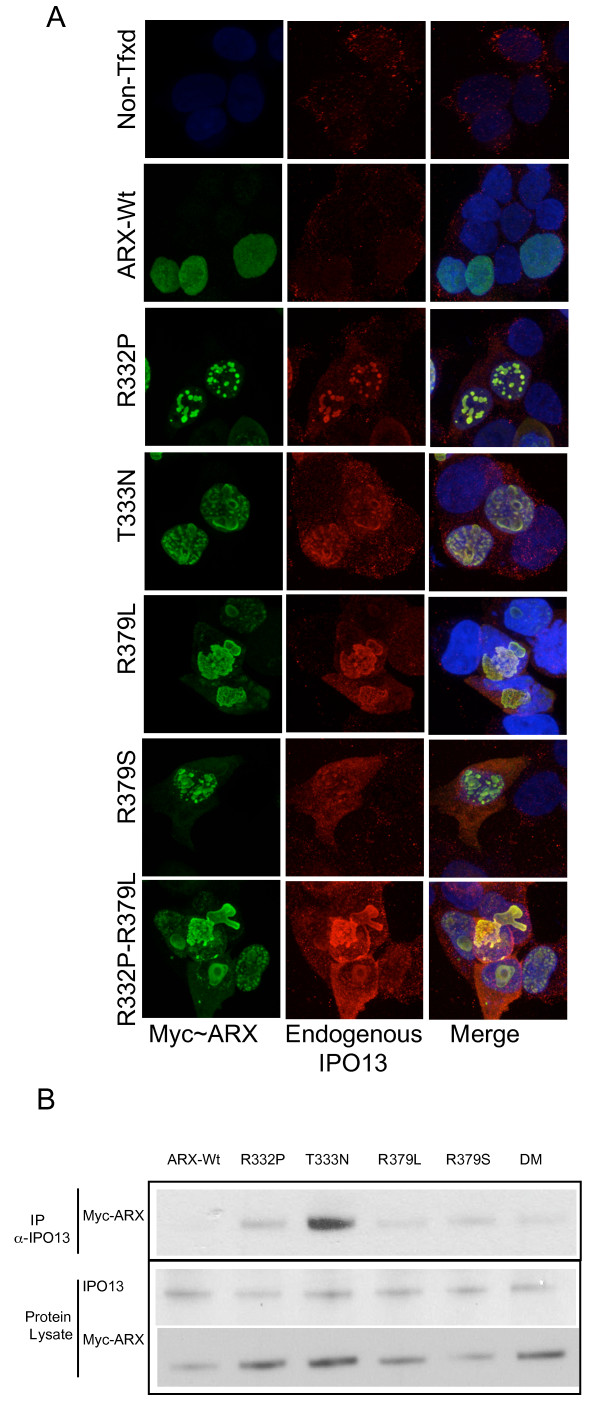
**Endogenous IPO13 co-localizes and co-immunoprecipitates with mutant ARX protein**. (A) Pictomicrographs showing sub-cellular localization of Wt and mutant Myc-ARX detected by anti-ARX antibody and FITC conjugated secondary antibody (left panel), endogenous IPO13 detected by anti-IPO13 antibody and Cy3 conjugated secondary antibody (middle panel) and merged image including blue DAPI stained nuclear material (right panel). Endogenous IPO13 co-localises with Myc-ARX mutant protein, including the double mutant R332P-R379L, in both nuclear and cytoplasmic aggregates, but does not co-localize with the ARX-Wt protein (lane 1). (B) HEK293T cells transfected with Myc-ARX-Wt or NLS mutant constructs were lysed and protein IP with antibodies against endogenous IPO13. Samples were loaded onto 4%-12% SDS-PAGE gels and analysed for the presence of co-IP proteins. IP with Goat anti-IPO13 antibody (top panel) and detection of Myc-ARX proteins bound to IPO13 by immunoblotting with mouse anti-Myc HRP conjugated antibody. All samples transfected with Myc-NLS mutant ARX showed a protein band of the correct size upon blotting with anti-Myc HRP conjugated antibody, with no band present in the ARX-Wt lane. Endogenous IPO13 (~108 kDa) and Myc-ARX (~62.2 kDa) are both present in protein lysates (bottom box). Scale bar = 10 μM.

HEK293T cells express detectable levels of endogenous IPO13 mRNA and protein. When we used an anti-IPO13 antibody the signal was localised as a diffuse punctate staining across the cytoplasm (Figure 4A top panel). In cells expressing mutant ARX, much of the endogenous IPO13 signal was sequestered with the ARX protein, with residual IPO13 signal in the cytoplasm (Figure 4A). This co-localization was observed in all cells expressing mutant ARX protein, including the protein with mutations in both NLS2 and NLS3 (Figure 4A bottom panel). In contrast, endogenous IPO13 protein did not co-localize with ARX-Wt (Figure 4A second panel), likely due to efficient re-cycling of IPO13 back to the cytoplasm after uncoupling from ARX in the RanGTP rich environment of the nucleus. We conclude that mutant ARX protein binding to endogenous full length IPO13 localizes in the same manner as if it were bound to the N-terminally truncated V5-IPO13 that cannot physically uncouple from its cargo. The interaction of endogenous IPO13 and mutant ARX protein was confirmed by Co-IP of Myc-ARX NLS mutant protein, but not the ARX-Wt protein from whole cell extracts using an antibody against endogenous IPO13 (Figure 4B, top panel).

### The binding of N-terminally truncated V5-IPO13 to ARX-Wt increases the proportion of cells with abnormal ARX protein localization

**Figure 5:**
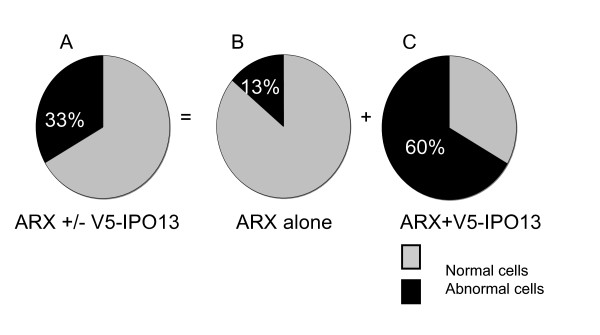
**ARX-Wt protein is abnormally localized when co-transfected with N-terminally truncated V5-IPO13**. HEK293T cells transfected with Myc-ARX-Wt and V5-IPO13 were assessed for localization of the resulting ARX protein, either normal nuclear localization (grey) or abnormal localization as inclusions or aggregates in the nucleus, with or without aggregates in the cytoplasm (black). (A) Combined count of cells transfected with ARX with or without V5-IPO13, (B) cells with only ARX expression and (C) cells with both ARX and V5-IPO13 expression.

When Myc-ARX-Wt is co-expressed with V5-IPO13, we measure an increased proportion of cells with abnormal localization of the ARX-Wt protein compared to cells expressing the ARX-Wt alone (Figure 5A). In cells transfected with ARX alone we find 13% of cells with abnormal ARX-Wt localisation (Figure 5B; in agreement with previous result; Figure 2D), but when the cells are co-expressing V5-IPO13 we note 60% of cells with abnormal localization of ARX-Wt protein (Figure 5C). In addition, these cells often had large or multiple aggregates within the nucleus and in some cases in the cytoplasm, reminiscent of the results for ARX-NLS mutant proteins (data not shown). This data clearly indicates that the binding of ARX-Wt protein to V5-IPO13 contributes to aggregation and mis-localization of the ARX.

### Potential pathogenic mechanism of NLS mutations in ARX

**Figure 6:**
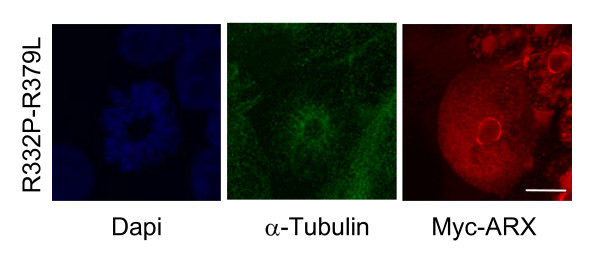
**Mitotic spindles disrupted by aggregates of NLS mutant ARX protein**. A representative pictomicrograph of a HEK293T cell transfected with mutant Myc-ARX undergoing DNA condensation stage of mitosis shows abnormal pattern of DNA condensation (DAPI) microtubule formation (alpha-tubulin) around a large aggregate of mutant ARX protein (Myc-ARX) with the remaining Myc-ARX signal localized diffusely throughout the cell interior. Scale bar = 10 μM.

In many cells with mutant ARX protein expression that were undergoing mitosis we noted the mitotic spindles were messy or even disrupted by aggregates of mutant protein (Figure 6). This raised the question of whether the formation of microtubules during cell division is affected by sequestration of ARX.

### Alpha Tubulin is a novel interacting protein partner of ARX

**Figure 7:**
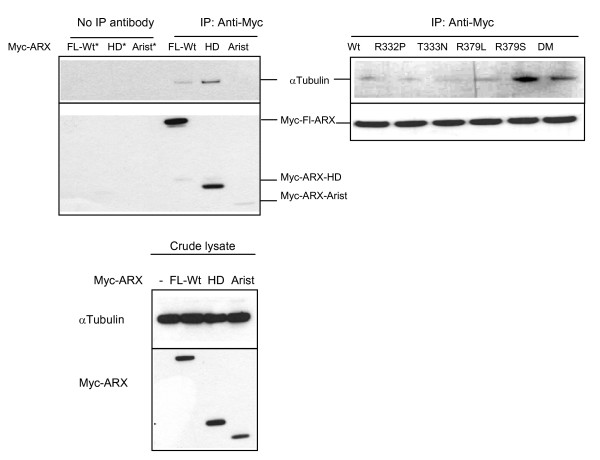
**Alpha tubulin co-immunoprecipitates with ARX in mammalian cells**. HEK293T cells transfected with Myc-ARX full length protein (FL-Wt), Myc-ARX-homeodomain (HD) or Myc-ARX-Aristaless (Arist) were lysed and proteins immunoprecipitated (IP) with mouse anti-Myc antibody. Samples were loaded on 4%-12% SDS-PAGE gels and analysed for the presence of endogenous alpha tubulin precipitated with Myc-ARX constructs by immunoblotting with rabbit anti-alpha tubulin antibody and goat anti-rabbit IgG conjugated to horseradish-peroxidase (HRP; top panel). Specific IP of Myc-ARX was achieved with no band present in samples from cells transfected with each of the Myc-ARX constructs but no IP antibody added (*). Myc-ARX full length (~62.2 kDa), Myc-ARX-HD (~19 kDa), Myc-ARX-Arist (~15 kDa) and endogenous alpha tubulin (~50 kDa) are present in crude protein lysates (bottom panel). HEK293T cells transfected with Myc-ARX NLS mutations were subjected to IP as above and alpha-tubulin was detected co-immunoprecipitating with all mutant ARX proteins (Left panel).

Interactions of ARX with cytoskeletal and other proteins were pursued using a GAL4-based yeast-2 hybrid screen. We identified alpha-tubulin (TUBA1A; NM_006009.2) and beta-tubulin (TUBB3; NM_006086.2) from a human fetal brain cDNA library. The bait protein used in this instance was the human ARX homeodomain (aa 303-431) fused to the GAL4 DNA binding domain (GAL4-DBD). This protein did not autoactivate the *HIS3, lacZ *or *URA3 *reporter genes upon transformation of MaV203 yeast (data not shown). Given the involvement of mutations in TUBA1A in phenotypes with lissencephaly and other similar clinical findings to the severe XLAG phenotype due to mutations in ARX we wanted to investigate the novel interaction between ARX and TUBA1A further. As there is a high level of similarity between members of the alpha-tubulin family and we predicted that ARX may interact not only with the brain specific TUBA1A but also with other members of the family. We confirmed this interaction in mammalian cells by Co-IP. Overexpressed Myc-tagged ARX was immunoprecipitated from whole cell extracts using a monoclonal anti-Myc antibody. Endogenous alpha-tubulin immunoprecipiated with Myc-ARX was detected by rabbit anti-alpha-tubulin antibody (raised against a common immunogen; aa 417 to 441 of TUBA1A, NP_00600.2) (Figure 7). When we tested the mutations in the NLS regions of ARX we observed Co-IP of alpha-tubulin for all samples, but not in parallel samples in which no IP antibody was added (Figure 7). Our data indicates that alpha-tubulin interacts with the homeodomain of ARX and this interaction is not abolished by any of the NLS mutations tested. Despite the interaction of ARX and alpha-tubulin by yeast-2 hybrid studies and Co-IP, in cells co-stained for Myc-ARX and alpha-tubulin we did not see any apparent co-localization of the two proteins. However, in mitotic cells expressing the mutant ARX protein we noted the condensation of DNA and the formation of mitotic spindles were often localized around aggregates of the mutant protein (Figure 6).

### Sequestration of mutant ARX and IPO13 may compromise mitosis

The effects of the sequestration of mutant ARX with IPO13 on mitosis and cell division were investigated at 24, 48 and 72 h post-transfection and compared with ARX-Wt. Co-staining of ARX (transfected cells expressing ARX protein), alpha-tublin (mitotic microtubules) and nuclear material (DAPI) was conducted to assist characterization of mitotic cells. At each time point cells were analysed for: (i) percentage of cells transfected; (ii) percentage of mitotic cells in both transfected and untransfected cells; and (iii) and the phase of mitosis for each cell undergoing division.

**Figure 8:**
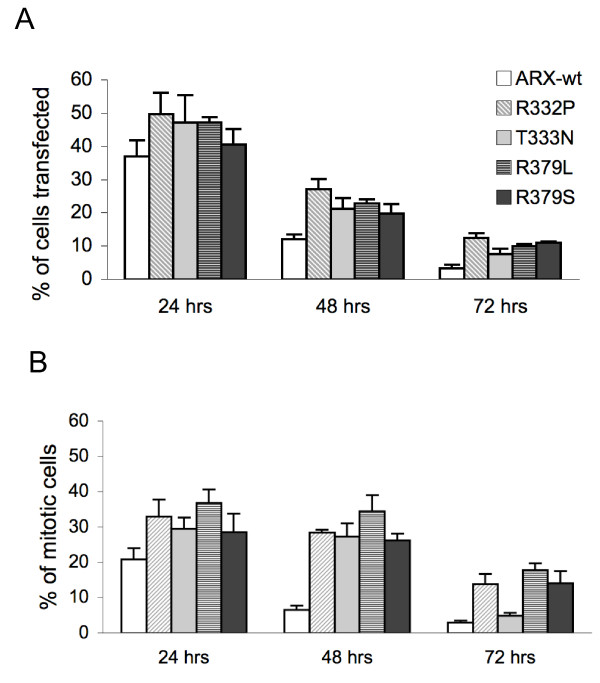
**Increased proportion of mitotic cells transfected with nuclear localization sequence (NLS) mutant ARX**. (A) The percentage of HEK293T cells transfected and (B) the percentage of mitotic cells transfected with Myc-ARX-Wt or NLS mutant constructs for 24, 48 and 72 h was determined from between 1200 and 4000 cells per sample, from at least three separate transfection reactions. Full-length Myc-tagged constructs transfected are indicated in the legend ARX Wt (open), R332P (vertical light grey stripes), T333N (grey), R379L (horizontal dark grey) and R379S (dark grey) with the time post-transfection indicated on the X-axis.

The percentage of cells expressing Myc-ARX were higher in cells transfected with mutant ARX compared to the ARX-Wt at all time points examined (Figure 8A). This difference was ~1.3-fold at 24 h, increasing to 1.6- to 2.2-fold by 48 h and between 2.3 (T333N) and 3.8 (R332P) fold by 72 h post-transfection. Although differences in the transfection efficiency may account for some of the initial difference at 24 h, it appears that the cells expressing mutant ARX protein persist in the population longer than cells expressing the ARX-Wt protein. The overall levels of mitosis (in both transfected and un-transfected cells) were consistently between 4%-5% of the total cell population, across all times and treatment groups (data not shown). However, there was a greater contribution to the mitotic pool in cells expressing mutant ARX protein compared to the ARX-Wt protein, increasingly prevalent with longer times post-transfection (Figure 8B). For example, although there were 1.3-fold more cells expressing R332P than ARX-Wt at 24 h, there were 1.6-fold more mitotic cells expressing the mutant protein compared to ARX-Wt protein. This difference was doubled at the 48 h time point, with 2.2-fold higher cells transfected but 4.4-fold more mitotic cells in cells with R332P expression compared to ARX-Wt. By 72 h post-transfection, although there were 3.9-fold more cells expressing R332P compared to ARX-Wt, the proportion undergoing mitosis was 4.7-fold higher in favour of the ARX mutant.

**Figure 9:**
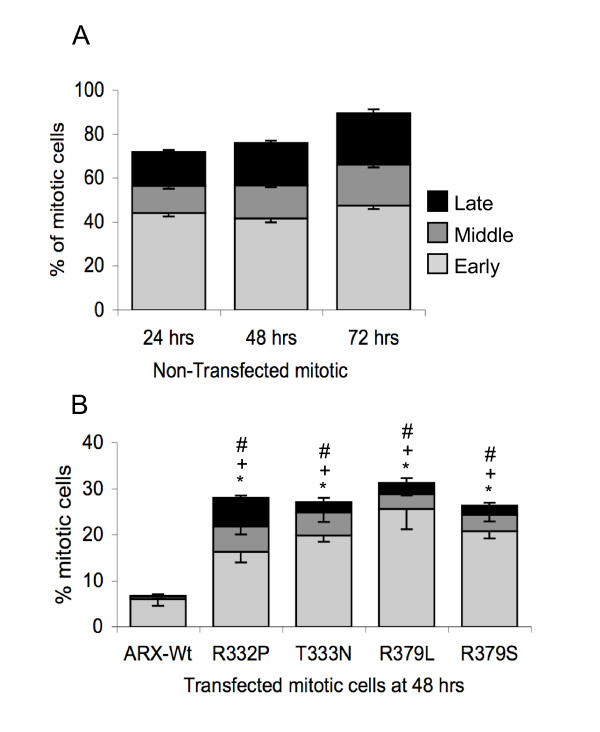
**Cells transfected with nuclear localization sequence (NLS) mutant ARX accumulate in early mitosis**. (A) The percentage of un-transfected HEK293T cells undergoing various phases of mitosis at 24, 48 and 72 h in culture (time indicated on the X-axis) were determined from between 1200 and 4000 cells per sample, from at least three separate transfection reactions. (B) The percentage of mitotic cells at the DNA condensation stage (early phase, light grey), mitotic spindle stage (middle phase, dark grey) or undergoing anaphase/telophase/cytokinesis (late phase, black) are shown for cells expressing Myc-ARX-Wt or NLS mutations 48 h post-transfection. Full-length Myc-tagged ARX used in each group is indicated on the X-axis. Differences in the percentage of cells undergoing each phase of mitosis at different times post-transfection were compared using a two-way ANOVA. *P *< 0.05 versus ARX-Wt for * early phase, + middle phase and # late phase.

In order to examine if there were any gross disturbances to the progression of the mitosis we scored each mitotic cell as being either: (1) early phase: representative of DNA condensation during prophase and prometaphase; (2) middle phase - DNA lining up across the mitotic spindle representative of metaphase; or (3) late phase - representative of anaphase through to telophase and cytokinesis. Each of these characteristic phases of cell division was readily delineated using normal light microscopy. Each mitotic cell was also scored as transfected or un-transfected. The data for cells without ARX expression undergoing mitosis for each time point was pooled. Not surprisingly, these cells accounted for an increasing proportion of mitotic cells across time points post-transfection, from 71% to 76% and 89% at 24, 48 and 72 h, respectively. Within each time point, over half of the mitotic cells without ARX expression were found to exist in the early phase with the remaining cells split between the middle and late phases (Figure 9A). Although the proportion of these mitotic cells increased with time post-transfection, the distribution across these phases was consistent.

When mitotic cells expressing mutant ARX proteins were analysed we saw an accumulation of cells undergoing mitosis compared to the cells expressing ARX-Wt (Figure 9B). In particular, there was a significant increase in the proportion of cells in the early phase of mitosis at 24, 48 and 72 h post-transfection in cells expressing the ARX NLS mutant proteins compared to cells expressing ARX-Wt (with the exception of T333N at 72 h). In cells expressing the ARX NLS mutations there was also a significant increase in the proportion of cells in the middle phase of mitosis at both 24 and 48 h post-transfection compared to the ARX-Wt protein. Interestingly, the percentages of cells in the late phase of mitosis expressing NLS mutations were also significantly higher than ARX-Wt at 48 h post-transfection (Figure 9B) but not at 72 h (data not shown). This indicates that cells expressing the ARX NLS mutation proteins are progressing through cell division at a slower rate compared to the ARX-Wt protein.

## Discussion

We have established that naturally occurring patient mutations leading to non-synonymous changes of single, specific residues of the NLS regions cause significant disruption to nuclear localization of the mutant ARX protein *in vitro*. These findings were consistent in all NLS2 and NLS3 mutations tested, indicating the mechanism involved may be similar for missense mutations in both NLS2 and NLS3 regions of the ARX homeodomain. Consistent with the severity of clinical outcomes, mutations leading to severe malformation phenotypes had the highest percentages of cells with abnormal mutant protein localization while the mutation causing ISSX but not brain malformation gave the smallest, but still significant, increase in cells with abnormal protein localization compared to the ARX-Wt protein. In contrast to the NLS mutations, another ARX mutation in the middle of the homeodomain, but outside of the NLS regions, did not disrupt the nuclear localization of the mutant protein with the subcellular localization of the mutant protein the same as the ARX-Wt protein.

The NLS regions flanking the ARX homeodomain are predicted to be important in the bi-partite binding to IPO13 [33,34]. We wanted to investigate if changing single, specific residues of the NLS regions would diminish or potentially abolish binding of the mutant NLS with IPO13. The interaction of importins and cargo proteins are often difficult to visualize or measure, as these interactions are highly transient with efficient recycling of the importin proteins back to the cytoplasm after delivery of the cargo to the nucleus. In order to capture this interaction, N-terminal truncated V5-IPO13 was engineered to enable binding to cargo and transport across the nucleus coupled with an inability to interact with RanGTP and dissociate upon reaching the nucleus [33]. Both the Co-IP and co-localization results indicate single amino acid substitutions in either the NLS2 or NLS3 regions of the ARX homeodomain did not abolish binding of the mutant ARX protein to N-terminally truncated V5-IPO13. Moreover, protein containing both R332P and R379L mutations was still able to bind to V5-IPO13.

Interestingly, binding of mutant ARX protein to endogenous IPO13 was indistinguishable from the binding to the N-terminally truncated IPO13 lacking the RanGTPase binding domain. The interaction of endogenous IPO13 with mutant ARX protein implies that the binding of these two proteins not only occurs but that this complex is either unable to correctly transport across the nuclear pore or unable to discharge the ARX cargo once inside the nucleus, or both. We contend that the inability of mutant ARX to uncouple from endogenous IPO13 contributes to the overall increase in cells with abnormal localisation of ARX protein. In support of this suggestion, co-transfection of ARX-Wt protein with the N-terminally truncated IPO13 led to a marked increase in the proportion of transfected cells with abnormal localization compared to cells transfected with ARX-Wt alone. Hence, disruption of normal nuclear delivery and distribution of mutant ARX protein may be due to a compromised ability of the mutant ARX-IPO13 complex to uncouple in the presence of RanGTP. Inadequate nuclear accumulation of a transcription factor due to impaired interaction with importin-β has been suggested as the pathogenic mechanism behind mutations in the C-terminal NLS of the *SRY *gene leading to XY genotype developing as females [38]. A similar scenario of inadequate accumulation and distribution of the mutant ARX protein within the nucleus might ultimately mimic complete absence of ARX and contribute to the catastrophic phenotypic consequences routinely observed in patients with these mutations. This prediction fits with the emerging genotype-phenotype pattern for mutations in ARX. In particular, naturally occurring mutations such as insertions [5,7,9,12], deletions [5,28-31], nonsense changes [30] and splice mutations [30] in ARX that result in protein truncation and loss-of function of the mature ARX protein invariably lead to severe phenotypes, including XLAG.

Both arginine residues examined in this study, R332 in NLS2 and R379 in NLS3 of ARX, are invariant in all 26 paired-type homeodomain proteins. Within this family of proteins these two basic residues are frequent sites of missense and nonsense mutations associated with a range of diseases. A mutation in the homologous NLS2 residue has been reported for *ALX4 *(MIM 605420) [39] and mutations in the homologous NLS3 residue have been identified in *CRX *(MIM 602225) [40], *OTX2 *(MIM 600037) [41] and *SHOX *(MIM 312865) [42,43]. Mutations in both residues corresponding to the R332 and R379 of ARX have been identified in *PAX3 *(MIM 606597) [44,45], *PAX6 *(MIM 607108) [46,47], *PITX2 *(MIM 601542) [48-50] and *PROP1 *(MIM 601538) [51-54]. A mutation in the conserved residue at position 50 of the paired-type homeodomain *SHOX *results in the abolition of DNA binding [55]. This residue corresponds to R379 of ARX. However, this residue is not part of the NLS region of the SHOX protein. When a residue within the NLS is mutated (R173C of SHOX) this change does not affect the DNA binding but instead disrupts the nuclear transport of SHOX. This may also be the case with the mutations tested in our study. There is no doubt that some of the missense mutations in the ARX homeodomain are likely to be important in either specificity of binding or in the actual binding to DNA. For example, the P353L mutation did not disrupt nuclear location of the resulting protein. Perhaps this mutation leads to the severe XMESID phenotype due to changes in binding to DNA targets. The recent identification of a specific transcription factor-binding site for Arx in a study ablating *Arx *in the sub-palluim of the mouse brain [56] will provide an important tool to investigate the potential mechanisms underlying the pathogenesis of mutations in the ARX homeodomain in the future.

A recent examination of heterozygous females from families with known mutations in *ARX *has highlighted the fact that in some cases mutations that disrupt ARX in females may have pathogenic consequences [32]. In particular, a number of females with the T333N and R379S mutations display mental retardation or learning disabilities, although other female carriers of the same mutation are phenotypically normal [30,32]. Our data indicates that in addition to a predicted loss of normal transcription factor activity of ARX, sequestration of mutant ARX with IPO13 may lead to a disruption of interactions with other protein partners, contributing to the disease phenotype. An emerging feature of many forms of lissencephaly and pachygyria is the potential disruption of key elements of microtubule behaviour. For example, one of the key genes mutated in lissencephaly is doublecortin (*DCX*). Mutations in *DCX *are clustered in two tubulin binding domains and impair the polymerization of microtubules and as such correct neuronal migration [57]. Interestingly, DCX does not bind to the tubulin heterodimer itself but acts to nucleate the microtubule growth and to stabilize microtubules. More recently, mutations in *TUBA1A *have been reported to cause lissencephaly with a distinct clinical presentation, ranging from perisylvian pachygyria in the less severe form, to posteriorly predominant pachygyria in the most severe form, in association with dysgenesis of the anterior limb of the internal capsule and mild to severe cerebellar hypoplasia [58]. A recurrent mutation in *TUBA1A *compromises the efficiency of *de novo *alpha/beta-tubulin heterodimer formation [59]. We have shown that ARX interacts with TUBA1A by Co-IP, but the two proteins do not appear to co-localize during normal cell growth. However, in cells expressing mutant ARX we observed a disruption of the mitotic spindle structures and an accumulation of cells in the early stages of mitosis. Hence, neurons, which normally express ARX during the development of the sequestration of mutant ARX, might compromise the interaction with TUBA1A, disrupt normal microtubule formation and subsequently retard efficient cell division.

We cannot rule out the possibility that sequestration of IPO13 in these cells may also contribute to pathogenesis. The dynamic cycling of Ran between the GTP and GDP bound forms is exquisitely controlled by specific regulators differentially localized within the cellular environment [60]. The RanGAP, which accelerates GTP hydrolysis, is cytoplasmic. The Ran guanine nucleotide exchange factor, also known as RCC1 (regulator of chromatin condensation 1), is located in the nucleus bound to chromatin. Hence, the levels of RanGTP increase in proximity to chromatin. Importin proteins bind and inhibit spindle accessory factors (SAF) everywhere in the mitotic cytosol, except in the vicinity of the chromosome. The high levels of RanGTP close to the chromatin relieve the inhibition of SAFs by importins and subsequently allow local spindle assembly. RanGTP binding to importin protein generates conformation change in the Importin molecule that in turn alters the binding site of the importin for the cargo proteins [37]. Therefore, IPO13 trapped in complex with mutant ARX may not be able to adequately bind SAFs and as such compromise spindle assembly. Samples of the cortex taken at autopsy from the patient with the R379L mutation were stained with neurofilament immunoperoxidase and, interestingly, a paucity of neurons was noted (D Coman, unpublished data). The authors could not rule out the contribution of perinatal ischemia to the histological findings. However, in light of our findings, it is interesting to speculate that disruption of the mitotic spindle due to sequestration of mutant ARX with IPO13 retards cell division, potentially contributing to a disruption in neuronal migration and correct lamination patterns of the brain leading to lissencephaly and associated clinical outcomes.

## Abbreviations

ARX: *Aristaless *related homeobox; HRP: horseradish-peroxidase; IP: Immunoprecipitation; IPO13: Importin 13; MRI: magnetic resonance imaging; NLS: Nuclear localization sequence; PBS: phosphate buffered saline; SAF: spindle accessory factor; TBS: tris buffered saline; TUBA1A: Tubulin A1A; XLAG: X-linked lissencephaly with ambiguous genitalia; XLID: X-linked intellectual disability; XMESID: X-linked myoclonic epilepsy with spasticity and intellectual disability.

## Competing interests

The authors declare that they have no competing interests.

## Authors' contributions

GM, D Coman, TK and GMM contributed samples and clinical data from affected individuals. D Cloosterman performed yeast-2 hybrid studies. CS, MHT and TF performed cloning, cell culture studies and functional assays and analysed the data. CS and JG conceived and designed the study. CS directed the study and wrote the first draft of the manuscript. All authors contributed to discussion of the results and manuscript preparation. All authors have read and approved the final manuscript.
